# The intensive care infection score – a novel marker for the prediction of infection and its severity

**DOI:** 10.1186/s13054-016-1366-6

**Published:** 2016-07-07

**Authors:** Patrick J. van der Geest, Mostafa Mohseni, Jo Linssen, Servet Duran, Robert de Jonge, A. B. Johan Groeneveld

**Affiliations:** Department of Intensive Care Medicine of the Erasmus Medical Center, ‘s Gravendijkwal 230, 3015 CE Rotterdam, The Netherlands; Faculty of Health Science, University of Medicine, Institute of Immunology, University Witten/Herdecke, Witten, Germany; Department of Intensive Care Medicine of the Maasstad Hospital, Rotterdam, The Netherlands; Department of Clinical Chemistry of the Erasmus Medical Center, Rotterdam, The Netherlands

**Keywords:** Critically ill, Infection, Septic shock, Biomarkers, PCT, ICIS

## Abstract

**Background:**

The prediction of infection and its severity remains difficult in the critically ill. A novel, simple biomarker derived from five blood-cell derived parameters that characterize the innate immune response in routine blood samples, the intensive care infection score (ICIS), could be helpful in this respect. We therefore compared the predictive value of the ICIS with that of the white blood cell count (WBC), C-reactive protein (CRP) and procalcitonin (PCT) for infection and its severity in critically ill patients.

**Methods:**

We performed a multicenter, cluster-randomized, crossover study in critically ill patients between January 2013 and September 2014. Patients with a suspected infection for which blood cultures were taken by the attending intensivist were included. Blood was taken at the same time for WBC, ICIS, CRP and PCT measurements in the control study periods. Results of imaging and cultures were collected. Patients were divided into groups of increasing likelihood of infection and invasiveness: group 1 without infection or with possible infection irrespective of cultures, group 2 with probable or microbiologically proven local infection without blood stream infection (BSI) and group 3 with BSI irrespective of local infection. Septic shock was assessed.

**Results:**

In total, 301 patients were enrolled. CRP, PCT and ICIS were higher in groups 2 and 3 than group 1. The area under the receiver operating characteristic curve (AUROC) for the prediction of infection was 0.70 for CRP, 0.71 for PCT and 0.73 for ICIS (*P* < 0.001). For the prediction of septic shock the AUROC was 0.73 for CRP, 0.85 for PCT and 0.76 for ICIS. These AUROC did not differ from each other.

**Conclusion:**

The data suggest that the ICIS is potentially useful for the prediction of infection and its severity in critically ill patients, non-inferiorly to CRP and PCT. In contrast to CRP and PCT, the ICIS can be determined routinely without extra blood sampling and lower costs, yielding results within 15 minutes.

**Trial registration:**

ClinicalTrials.gov identifier: ID NCT01847079. Registered on 24 April 2013.

## Background

Infection is probable or can be proven in approximately 50 % of critically ill patients with suspected infection [1, 2]. This could lead potentially to overtreatment with empiric antibiotics [1, 2]. Moreover, infection diagnostics are often delayed because it takes 48 h at the minimum for cultures to become positive and thereby to prove a clinically suspected infection. Hence, there is a continuing need for fast and accurate biomarkers of infection, which may help to predict infection, its invasiveness and severity, and may guide empiric antibiotic treatment in the future [3]. We believe that prediction of infection is more helpful in patient management than prediction of sepsis, because the majority of critically ill patients have two or more criteria of the systemic inflammatory response syndrome (SIRS) as criteria for sepsis, irrespective of infection.

Commonly applied biomarkers include C-reactive protein (CRP) and procalcitonin (PCT), but predictive values vary among studies [3–11]. A new biomarker is the intensive care infection score (ICIS), which is composed of five blood-cell-derived parameters characterizing the early innate immune response and is routinely obtainable in blood samples sent to the laboratory for cell counts. The ICIS has been retrospectively evaluated in two pilot studies of 70 and 172 patients, respectively, suggesting it has potential predictive value for infection [12, 13].

We therefore performed a prospective study on the predictive value of ICIS for probable or proven infection in critically ill patients with suspected infection, and compared its performance with that of the white blood cell count (WBC), CRP and PCT levels. We hypothesized that in the critically ill patient with suspected infection the diagnostic accuracy of the simply obtainable ICIS is at least equivalent in this respect to WBC, CRP and PCT, without requiring extra blood sampling.

## Methods

### Study design and patients

The ICIS study is an add-on non-interventional study of patients who had been enrolled into a prospective, cluster-randomized, crossover trial, involving both intensive care units (ICUs) of the Erasmus Medical Center Rotterdam and both ICUs of the Maasstad hospital Rotterdam. The ICUs were stratified and randomized by treatment regimen into a control group (standard of care) and an intervention group. In the intervention arm, blood culturing for a suspected infection was guided by PCT measurements. The acronym for PCT-guided blood culturing in the intensive care, ProBIC, was used for this study and results will be reported later. The trial was conducted between January 2013 and September 2014. The ICU of the Erasmus Medical Center is a tertiary care mixed medical-surgical ICU with approximately 2000 admissions per year. The ICU of the Maasstad hospital is a secondary care mixed medical-surgical ICU with 1200 admissions per year.

The trial was conducted in accordance with the ethical principles decreed by the Declaration of Helsinki and in compliance with International Conference on Harmonization Good Clinical Practice Guidelines. The institutional review board (IRB) or the independent medical ethical committee at each of the investigational centers (Medisch Ethische commissie Maasstad ziekenhuis, Rotterdam, Nederland and Medisch Ethische commissie Erasmus Medisch Centrum, Rotterdam, Nederland) reviewed and approved the protocol, amendments and informed consent document. The medical ethical committee of the Erasmus Medical Center finally approved the study (MEC 2011-505). The trial was registered at ClinicalTrial.gov (protocol ID NCT01847079) on 24 April 2013. All patients or their proxy provided written informed consent prior to study inclusion, at ICU admission.

Inclusion criteria were age above 18 and below 80 years and the clinical suspicion of infection, for which the attending intensivist established a medical need for blood culture. Suspicion of infection included but was not limited to increased body temperature above 38.3 °C (tympanic temperature), chills, progressive leukocytosis, increased CRP, increasing consolidation on chest radiography or other imaging of potential infection sources. It was possible for each patient to be included more than once, but in the current study we only analyzed the first time that blood was sampled for culture. Patients were excluded if they were pregnant, had neutropenia (defined as leukocyte count less than 0.5 × 10^9^/L), used immunosuppressive or immunostimulatory therapy, or had a predetermined illness with death expected within 24 h. Patients were not included if blood cultures were performed as part of a standard protocol (such as patients with veno-venous or veno-arterial extracorporeal membrane oxygenation (ECMO)) or were performed to check the effectiveness of treatment (such as in endocarditis), unless the blood culture was done because of suspicion of infection. The ICUs switched the allocated regimen every 3 months, so that there were six 3-month episodes of standard care in which 774 patients were eligible for inclusion, and 473 patients were excluded (5 patients who were ≤18 years of age; 63 with neutropenia (<0.5 × 10^4/^L); 35 with uncontrolled malignancy; 256 on immunosuppressive medication; 22 who were expected to die within 24 h; and 92 without informed consent). Data for the ICIS study were thus collected in 301 patients in the control arm (six 3-month episodes) of the ProBIC study.

### Study protocol, data collection and assays

Baseline demographic data and clinical variables were recorded on the day of inclusion, and included age, sex, comorbidity, reasons for admission, use of antibiotics including selective decontamination of the digestive tract (SDD), antifungal treatment, steroids, immunosuppressive medication, immune status and recent surgery. The treatment received during ICU stay was also recorded and included mechanical ventilation, renal replacement therapy, total parenteral nutrition, arterial and central venous catheters, and the use of vasopressor or inotropic medication. The acute physiology and chronic health evaluation II (APACHE II) and the sequential organ failure assessment (SOFA) score were recorded at admission. The length of ICU and hospital stay and vital outcomes were recorded for up to 90 days after inclusion.

At the same time that blood was taken for culture, blood samples were taken for determination of WBC, CRP, PCT, and ICIS (day 0). Blood for similar measurements (except for PCT) was taken in the morning on the two following days (days 1 and 2). Treating physicians and investigators were blinded to the PCT and ICIS measurement results. Also the outcome adjudicators that decided presence or absence of infection were blinded to the biomarker results. Two sets of blood cultures were taken and directly sent to the department of medical microbiology. The set taken for blood culture consisted of one aerobic and one anaerobic bottle (BD Bactec™, Franklin Lakes, NJ, USA), which contain resin to enhance recovery of organisms. The samples were incubated for a 7-day period in an automatic analyzer (BD Bactec™) that automatically demonstrates the time to positive blood culture in the case of positive bacterial or fungal growth. Gram strains were performed, and the organisms were cultured on agar plates after identification of growth using the VITEK® 2 (Biomerieux, Marcy l’Etoile, France).

Blood for the WBC and ICIS measurement was obtained in a K3EDTA tube. Both the WBC and ICIS parameters were measured on a modified fluorescence flow hematology analyzer with fully automated gating (Sysmex, Kobe, Japan) [14]. The ICIS was measured promptly after collection but within a maximum of 24 h. The ICIS score is composed of five blood-cell-derived parameters that characterize the innate immune response [15–19]. The five parameters include the mean fluorescence intensity of mature (segmented) neutrophils, the difference in hemoglobin concentration between newly formed and mature red blood cells, the total segmented neutrophil count, the antibody secreting lymphocytes, and the accurate immature granulocytes count, as previously described [12]. Each parameter is available from a standard routine method and can be measured within 1 minute without sample preparation on a modified fluorescence flow hematology analyzer with fully automated gating (Symex) [12]. The methodology is based on routine hematology fluorescence flow cytometry using different fluorescence reagents for mainly nucleic acids, and specifically designed blood cell membrane surfactant reagents generating information about cell shape and the formation of bioactive lipids from cell membranes [12]. Side and forward scatter light are used to determine the intracellular structure and size of blood cells [12]. By adding all weighting values for all five parameter components, the maximum possible ICIS is 20. Serum CRP (turbidimetric assay) and PCT (electrochemiluminescence BRAHMS immunoassay) measurements were routinely performed using a Cobas 8000 platform (Roche, Almere, Netherlands). Blood for PCT measurement was sampled in a z serum clot activator tube.

### Definitions

After completion of the study, the investigators decided whether an infection was present from days 0–2, on the basis of the available imaging and culture results. The outcome adjudicators were blinded to all biomarkers. Source and likelihood of infection were based on criteria defined at the International Sepsis Forum Consensus Conference [20]. Culture results were analyzed within a 48-h window from before and after taking blood cultures. The causative microorganisms were recorded. BSI was defined as a positive blood culture with a recognized pathogen except for skin contaminants [20, 21]. In the case of skin contaminants, BSI was identified if at least two blood cultures drawn on separate locations were positive [20, 21]. Patients were divided into groups according to increasing likelihood of infection and invasiveness of associated microorganisms that was suggestive of increasing severity: group 1 without infection or with possible infection irrespective of cultures; group 2 with probable (irrespective of cultures) or proven local infection (with positive cultures of a causative microorganism) without BSI; and group 3 with BSI irrespective of local infection. SIRS was defined as two or more of the following criteria: (1) body temperature >38 °C or <36 °C; (2) WBC (>10,000/μL), leukopenia (<4,000/μL), or >10 % bands; (3) heart rate >90 beats/minute; and (4) respiratory rate >20 breaths/minute or mechanical ventilation, for values at day 0. When SIRS and a probable/proven infection (groups 2 or 3) were present, patients were classified as having sepsis. Shock was defined as acute circulatory failure characterized by persistent systolic arterial pressure <90 mm Hg or mean arterial pressure (MAP) <70 mm Hg for at least 1 h despite adequate fluid resuscitation or requirement of vasopressor support to maintain MAP, at day 0. In the presence of sepsis, shock was defined as septic shock.

### Statistical analysis

This was performed using SPSS version 23 (SPSS inc., Chicago IL, USA) and using R package. Data are expressed as median (interquartile range) or as number of patients (percentage) where appropriate. Most data were distributed non-normally (Kolmogorov-Smirnov test *P* < 0.05). Group (>2) differences were evaluated using the Kruskal-Wallis test or chi-square (*X*^2^) test, for continuous and categorical data, respectively. The Mann-Whitney *U* test and Fisher exact test were used to compare two groups. To evaluate predictive values we calculated the areas under the receiver operating characteristic curves (AUROC) for day 0 values. For the predictive values of sepsis and septic shock we used the values for day 0. We consider an AUROC >0.70 as clinically relevant [22]. The optimum cutoff value was calculated on the basis of the highest sensitivity and specificity combined (Youden index). Positive and negative predictive values were calculated. To correct for multiple testing we set the level of statistical evidence at *P* ≤ 0.01. Exact *P* values >0.001 are given.

## Results

### Patient characteristics

Table 1 describes the baseline characteristics of the 301 patients enrolled: 149 patients (group 1) had no infection and 152 patients (groups 2 + 3) had a probable or proven infection. Patients with a probable or proven infection were older and more often had a history of cancer, cardiac disease or gastrointestinal problems. Mechanical ventilation or renal replacement therapy was more often used in patients with a probable or proven infection. All patients with a probable or proven infection were on antibiotics. No difference was seen in 28-day or 90-day mortality or in the length of ICU or hospital stay.

**Table 1:** Baseline demographic and clinical characteristics

	Group 1	Groups 2 + 3	*P*
	(n = 149)	(n = 152)	
Age (years)	57 (24)	62 (19)	0.01
Gender (male)	100 (67)	105 (69)	0.72
APACHE II score	22 (10)	22 (8)	0.92
APACHE IV score	63 (38)	60 (34)	0.49
SOFA score	7 (7)	8 (6)	0.06
Comorbidity			
Neurologic	39 (26)	41 (27)	0.88
Cardiac	40 (27)	58 (38)	0.04
Pulmonary	28 (19)	38 (25)	0.25
Gastrointestinal	39 (26)	55 (36)	0.05
Renal	15 (10)	24 (16)	0.14
DM II	22 (15)	33 (22)	0.13
Cancer	22 (15)	46 (30)	0.002
Autoimmune	7 (5)	8 (5)	0.97
Reasons for ICU admission			<0.001
Suspected infection	23 (15)	69 (45)	
Respiratory failure	24 (16)	33 (22)	
Renal failure	0 (0)	1 (1)	
Liver failure	5 (3)	3 (2)	
Neurology	31 (21)	8 (5)	
CPR	9 (6)	5 (3)	
Shock	10 (7)	3 (2)	
Trauma	13 (9)	5 (3)	
Postoperative	34 (23)	25 (17)	
Treatment on ICU			
Antibiotics	140 (94)	152 (100)	0.02
Norepinephrine	109 (73)	128 (84)	0.03
Dobutamine	18 (12)	17 (11)	0.80
TPN	39 (26)	50 (33)	0.20
Mechanical ventilation	133 (89)	125 (82)	0.06
Renal replacement therapy	15 (10)	52 (34)	<0.001
Length of ICU stay (days)	9 (17)	11 (17)	0.49
Length of hospital stay (days)	22 (34)	26 (36)	0.13
Mortality day 28	42 (28)	58 (38)	0.09
Mortality day 90	98 (36)	68 (45)	0.13

Numbers (percentage) or median (interquartile range), where appropriate. Abbreviations: *APACHE II* acute physiology and chronic health evaluation II, *CPR* cardiopulmonary resuscitation, *DM II* diabetes mellitus type II, *ECMO* extracorporeal membrane oxygenation, *SOFA* sequential organ failure assessment score, *TPN* total parenteral nutrition

### Source of infection and microbial species

The abdomen and lungs were the most frequent source of infection (Table 2). Gram-positive pathogens were mostly cultured, followed by Gram-negative pathogens, fungi and viruses (Table 2).

**Table 2:** Infection characteristics

	Group 1	Group 2	Group 3	*P*
	(n = 149)	(n = 91)	(n = 61)	
Source of infection				0.01
Pulmonary	-	44 (48)	15 (25)	
Abdominal	-	30 (33)	29 (47)	
Urogenital	-	8 (9)	2 (3)	
Neurologic	-	2 (2)	2 (3)	
Soft tissue/bones	-	7 (8)	10 (17)	
Blood and catheter	-	0	3 (5)	
Gram strain				0.01
Gram-negative	-	30 (33)	16 (26)	
Gram-positive	-	27 (30)	36 (59)	
Type of microorganism				0.15
Staphylococci	-	15 (16)	17 (28)	
Streptococci	-	12 (13)	19 (31)	
Enterobacteriaceae	-	26 (29)	15 (25)	
Pseudomonas	-	4 (4)	1 (2)	
Fungi	-	12 (13)	6 (10)	
Viral	-	5 (5)	3 (5)	
Biomarkers				
SIRS	146 (98)	90 (99)	58 (95)	0.94
Septic shock	-	12 (13)	9 (15)	0.78
Temperature (°C)	38.1 (1.5)	38.3 (1.7)	38.0 (1.5)	0.94
Heart rate (beats/minute)	105 (29)	109 (37)	112 (39)	0.15
Respiratory rate (breaths/minute)	28 (13)	29 (17)	29 (17)	0.61
WBC day 0 (10^9^/L)	12.4 (7.8)	14.4 (12.9)	13.9 (12.2)	0.63
WBC day 1 (10^9^/L)	11.7 (8.1)	13.9 (13.1)	13.4 (11.3)	0.47
WBC day 2 (10^9^/L)	12.1 (7.2)	14.4 (9.8)	14.8 (15.0)	0.19
CRP day 0 (mg/L)	84 (109)	163 (156)	167 (161)	<0.001
CRP day 1 (mg/L)	88 (131)	156 (156)	197 (154)	<0.001
CRP day 2 (mg/L)	82 (141)	131 (136)	180 (172)	<0.001
PCT day 0 (μg/L)	0.65 (2.30)	2.71 (9.88)	4.13 (38.0)	<0.001
ICIS day 0	3 (3)	6 (5)	6 (5)	<0.001
ICIS day 1	3 (3)	6 (4)	6 (4)	<0.001
ICIS day 2	4 (4)	6 (4)	6 (4)	<0.001

Numbers (percentage) or median (interquartile range), where appropriate. Group 1: no infection; Group 2: local infection without blood stream infection; Group3: blood stream infection. Abbreviations: *CRP* C-reactive protein, *ICIS* intensive care infection score, *PCT* procalcitonin, *SIRS* systemic inflammatory response syndrome, *WBC* white blood cells

### Biomarkers

Table 2 shows the infection markers according to invasiveness of infection. Most patients had SIRS on day 0, so that the patients with infection in groups 2 and 3 had mostly sepsis. CRP, PCT and ICIS were increased on days 0–2 in patients with infection as compared to those without infection. In contrast to PCT, there was no difference in CRP and ICIS between groups 2 and 3. The CRP, PCT and ICIS were increased in patients with septic shock (Table 3).

**Table 3:** Septic shock

	No (n = 280)	Yes (n = 21)	*P*
Temperature (°C)	38.2 (1.5)	38.2 (1.6)	0.75
Heart rate (beats/minute)	108 (33)	119 (59)	0.04
Respiratory rate (breaths/minute)	28 (15)	29 (13)	0.85
WBC day 0 (10^9^/L)	12.8 (9.5)	12.5 (17.6)	0.68
WBC day 1 (10^9^/L)	12.4 (9.5)	14.1 (20.4)	0.60
WBC day 2 (10^9^/L)	12.5 (9.4)	14.6 (14.4)	0.41
CRP day 0 (mg/L)	107 (144)	234 (182)	<0.001
CRP day 1 (mg/L)	124 (147)	327 (160)	<0.001
CRP day 2 (mg/L)	107 (144)	244 (205)	<0.001
PCT day 0 (μg/L)	1.15 (6.1)	32.2 (94.0)	<0.001
ICIS day 0	4 (5)	9 (6)	<0.001
ICIS day 1	4 (4)	8 (7)	<0.001
ICIS day 2	5 (5)	7 (6)	0.03

Median (interquartile range). Abbreviations: *CRP* C-reactive protein, *ICIS* intensive care infection score, *PCT* procalcitonin, *WBC* white blood cells

### Predictive values

**Fig. 1:**
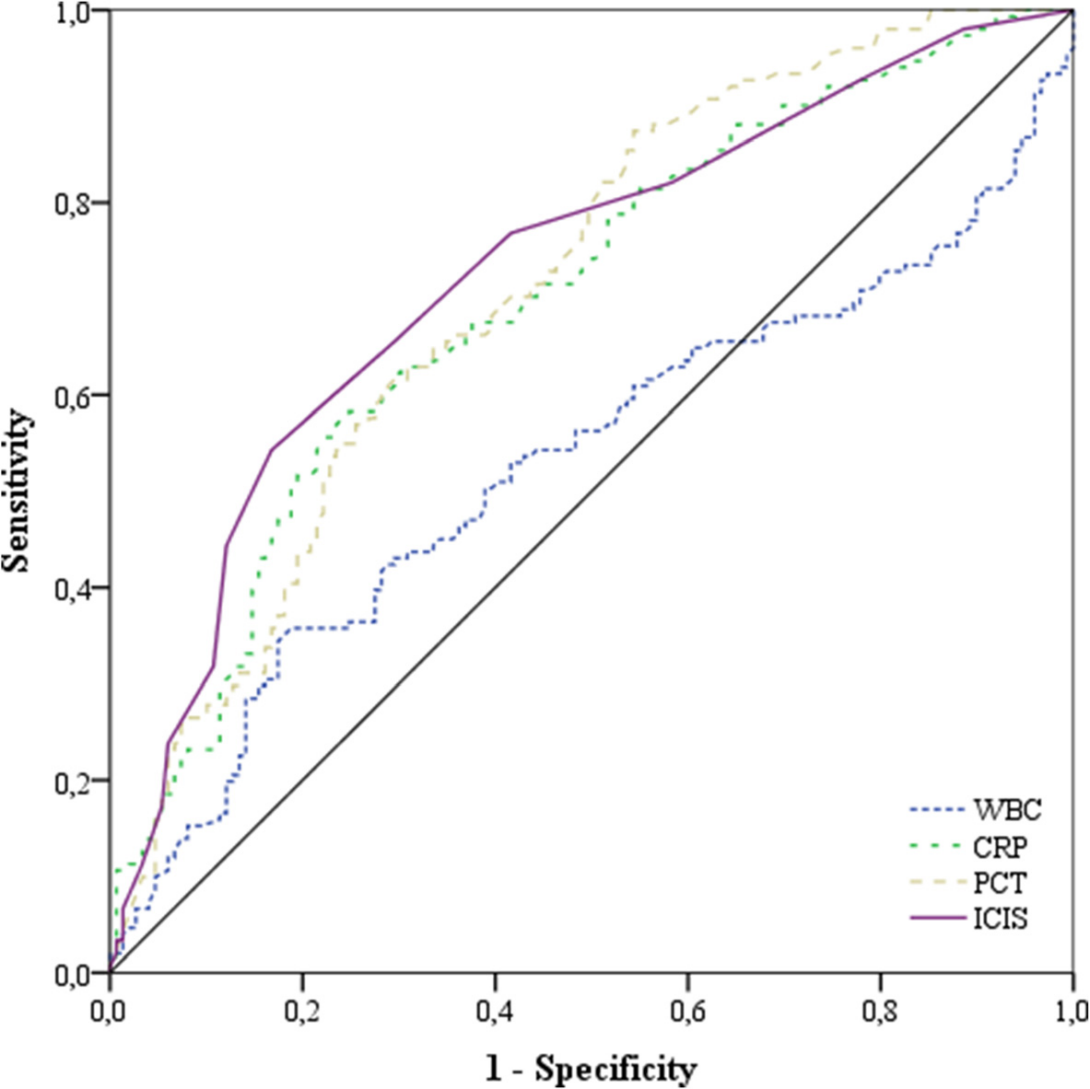
Area under the receiver operating characteristic curve for the four biomarkers for the prediction of infection: for white blood cell count (*WBC*) 0.53, for C-reactive protein (*CRP*) 0.70, for procalcitonin (*PCT*) 0.71, and for intensive care infection score (*ICIS*) 0.73

**Fig. 2:**
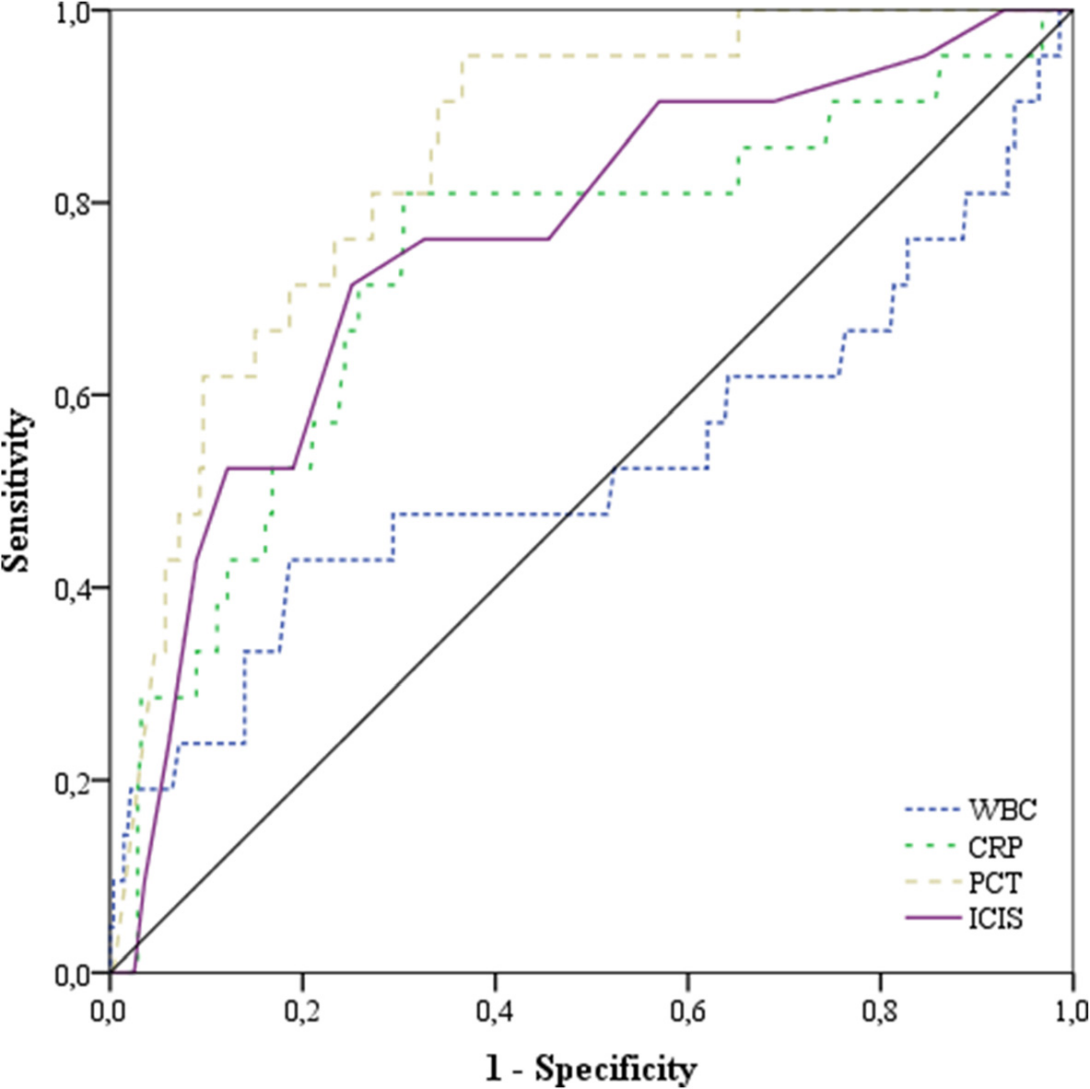
Area under the receiver operating characteristic curve for the four biomarkers for the prediction of septic shock: for white blood cell count (*WBC*) 0.53, for C-reactive protein (*CRP*) 0.73, for procalcitonin (*PCT*) 0.85 and for intensive care infection score (*ICIS*) 0.76

The AUROC for the prediction of infection (groups 2 + 3 vs group 1) on day 0 was similar for CRP, PCT and ICIS (Table 4, Fig. 1). At a cutoff ≥7, the positive predictive value of ICIS was >80 % and at a cutoff ≤1 the negative predictive value of ICIS was >80 %. Otherwise, the AUROC for ICIS did not differ from that of any other biomarkers, including PCT, expect for that of WBC (*P* < 0.001). The highest AUROC for the prediction of septic shock was for PCT (AUROC 0.85, *P* < 0.001), but this was not significantly different to the AUROC for ICIS (AUROC 0.76, *P* < 0.001), whereas the AUROC for CRP was 0.73 (*P* < 0.001) and the AUROC for WBC was 0.53 (*P* = 0.68) (Fig. 2).

**Table 4:** Receiver operating characteristic curve analysis to determine the optimum cutoff value of the different biomarkers on day 0 for the prediction of infection (groups 2 + 3)

	Parameters						
Biomarkers	AUROC (95 % CI)	*P*	Cutoff	Sensitivity	Specificity	PPV	NPV
WBC (10^9^/L)	0.53 (0.46, 0.60)	0.38	12.9	0.54	0.54	0.55	0.54
CRP (mg/L)	0.70 (0.64, 0.76)	<0.001	111	0.65	0.64	0.65	0.64
PCT (μg/L)	0.71 (0.66, 0.77)	<0.001	1.41	0.65	0.66	0.66	0.65
ICIS	0.73 (0.67, 0.79)	<0.001	5	0.66	0.71	0.70	0.67

Abbreviations: *AUROC* area under the receiver operating characteristic curve, *CI* confidence interval, *CRP* C-reactive protein, *ICIS* intensive care infection score, *PCT* procalcitonin, *PPV* positive predictive value, *NPV* negative predictive value, *WBC* white blood cells. The AUROC for ICIS differed from that for WBC (*P* < 0.001)

## Discussion

This study evaluated the predictive values of ICIS to discriminate between non-infectious systemic inflammation and infection (mostly sepsis) in critically ill patients with a suspicion of infection. The data suggest that ICIS is a useful marker to predict probable or proven infection and its severity and is non-inferior in this respect to CRP and PCT.

In the current study the frequency of probable or proven infection was 56 % of patients when an infection was suspected, which is comparable with the reported frequency of 51–58 % in large studies on the epidemiology of sepsis in the ICU [23]. The lung and abdomen were the most common origin of sepsis, followed by infections of soft tissues, as described before [24]. A large recently performed study showed that Gram-negative bacteria were isolated in 62 % of patients with sepsis who had positive cultures, Gram-positive bacteria in 47 %, and fungi in 19 % [23]. The results are in contrast with our study, which suggests that Gram-positive isolates are most likely to cause infection. The difference can be explained by the fact that we use SDD in our ICUs, which is known to eliminate Gram-negative bacteria and fungi from the digestive tract [25]. Blood cultures are typically positive in approximately one third of the patients with sepsis, in line with the incidence of 20 % in this study [24]. The overall ICU and hospital mortality rates were 28 and 37 %, respectively. The results are comparable with the reported rates in a European multicenter study of critically ill patients with sepsis [26].

In the current study the predictive values of WBC and CRP for infection and sepsis are comparable with those identified in previous studies, in which a low AUROC of 0.55–0.66 for WBC (sensitivity 65–91 %; specificity 35–54 %) and an intermediate AUROC of 0.64–0.77 for CRP (sensitivity 82–100 %; specificity 40–64 %) was reported [4, 5, 10, 11]. Large reviews report an AUROC of 0.78–0.81 for PCT (sensitivity 42–100 %; specificity 48–100 %), comparable to our study [7, 9]. The reported predictive value of ICIS in this study is lower compared with two previous studies, in which AUROC of 0.79 (sensitivity 70 %; specificity 79 %) and 0.85 (sensitivity 80 %; specificity 75 %) were reported, respectively [12, 13]. Both studies investigated a relatively small number of patients or investigated postoperative critically ill patients only [12, 13]. They were pilot studies to define the cutoff values of ICIS as a marker of infection in critically ill patients and recommended determination of the suitability and effectiveness of this score in a prospective trial [12, 13].

Using ICIS has several advantages over using CRP or PCT. First, no extra blood needs to be taken since the ICIS can be measured from the same K3EDTA tube that is used for the WBC measurement, thereby allowing routine daily measurements. Second, lower costs are involved because the ICIS measurement is performed on the same machine used for a WBC measurement. The major limitation is that in our study the predictive values of biomarkers including ICIS was not very high. Nevertheless, a high ICIS increases the likelihood of infection when suspected and a low ICIS decreases it. This may help the clinician in ordering extra tests or starting empiric antibiotics. The predictive value of ICIS for infection and septic shock is comparable with that of the percentage of immature granulocytes as assessed in a relatively small study in critically ill patients [27]. Both the percentage of immature granulocytes and ICIS can be obtained routinely without extra blood sampling or cost, though the current study focused on the diagnostic accuracy of ICIS and not its feasibility or cost-effectiveness. For future use the ICIS is expected to prove more robust.

## Conclusions

In conclusion, the present study suggests that ICIS is a novel and potentially useful predictor of infection and sepsis in critically ill patients with a suspected infection. The ICIS score can be collected routinely without extra blood sampling and with lower costs, yielding results within 15 minutes.

## Key messages

- The ICIS score is composed of five blood-cell-derived parameters that characterize the innate immune response
- The ICIS score is elevated in patients with probable or proven infection in the critically ill, similar to CRP and PCT
- The ICIS score is a novel and potentially useful predictor of infection and sepsis in critically ill patients
- The ICIS score can be collected routinely without extra blood sampling and at a lower cost as compared to CRP and PCT determinations

## Abbreviations

APACHE II, acute physiology and chronic health evaluation II; AUROC, area under the receiver operating characteristic curve; BSI, blood stream infection; CI, confidence interval; CPR, cardiopulmonary resuscitation; CRP, C-reactive protein; DM II, diabetes mellitus type II; ECMO, extracorporeal membrane oxygenation; ICIS, intensive care infection score; ICU, intensive care unit; IRB, institutional review board; MAP, mean arterial pressure; NPV, negative predictive value; PCT, procalcitonin; PPV, positive predictive value; SDD, selective decontamination of the digestive tract; SIRS, systemic inflammatory response syndrome; SOFA, sequential organ failure assessment score; TPN, total parenteral nutrition; WBC, white blood cells
